# Stress and distress in healthcare students: protective roles of social support, student community and meaningfulness of studying

**DOI:** 10.5116/ijme.674f.2679

**Published:** 2024-12-23

**Authors:** Katariina Pänkäläinen, Taina Hintsa, Marko Elovainio, Eeva Pyörälä, Tiina Paunio

**Affiliations:** 1 University of Eastern Finland, Department of Educational Sciences and Psychology, Finland; 2 University of Helsinki, Medical Faculty, Department of Psychology, Finland; 3 University of Helsinki, Center for University Teaching and Learning, Finland; 4 University of Helsinki, Medical Faculty, Clinicum, Finland

**Keywords:** Perceived stress, psychological distress, social support, sense of community, meaningfulness of studying

## Abstract

**Objectives:**

To explore association between perceived stress and psychological distress (depressive symptoms and anxiety), and the stress-buffering effects of social support (parents, partners, friends, peers, teachers, social media), sense of community belonging and meaningfulness of studying.

**Methods:**

A cross-sectional study was conducted in 2018 using a convenience sample of 800 healthcare students from the University of Helsinki, Finland. Participants completed an online survey. Logistic regression analyses were performed using the PROCESS Macro to explore the relationship between perceived stress and psychological distress, and the moderator effects.

**Results:**

Perceived stress was associated with depressive symptoms and anxiety. Perceived stress had significant interactions with parent (B =-.03, t_(783)_ = -2.4, p < .001), partner (B = -.05, t_(783) _= -4.3, p < .001) and peer support (B=-.04, t_(783) _= -3.0, p < .001), sense of community belonging (B=-.06, t_(783)_ = -2.7, p < .01) and meaningfulness of studying (B=-.12, t_(783)_ = -4.5, p < .001) in predicting depressive symptoms, and with parent (B = -.05, t_(783)_= -3.8, p < .001), partner (B = -.03, t_(783)_ = -2.2, p < .05) and peer support (B=-.05, t_(783)_ =-3.5, p < .001), sense of community belonging (B=-.05, t_(783)_=-2.5, p < .01) and meaningfulness of studying (B = -.08, t_(783)_ = -3.0, p < .01) in predicting anxiety. Perceived stress had weaker effects on depressive symptoms and anxiety at higher levels of support, sense of community belonging and meaningfulness of studying.

**Conclusions:**

Support from parents, romantic partners and peers, sense of community belonging and meaningfulness of studying may buffer the negative psychological outcomes of perceived stress. Promoting social support, sense of community and the meaningfulness of studying can help prevent psychological distress in healthcare students. Longitudinal research and further investigation on factors related to sense of community belonging and meaningfulness of studying are warranted.

## Introduction

Previous studies have shown that students in healthcare fields experience substantial stress during their studies.^1^^-^^4^ From a psychological perspective, stress refers to feelings of emotional pressure and strain, and it often results from a discrepancy between personal resources and environmental demands.^5^ For healthcare students, these demands can include anything from regular study-related activities, like exams and essays, to witnessing human suffering and even death.^6^^-^^9^Persistent, poorly managed stress and associated changes in cognitive, parasympathetic and immunological functioning may lead to the development of psychological distress, such as depressive symptoms and anxiety.^10^^-^^13^ Psychological distress can, in turn, impair academic performance^14^^,^^15^ and increase the risk for future ill-being and burnout.^16^ Identifying factors that help students manage the stressors of their education is therefore highly important when training future healthcare professionals.

Social support has been shown to buffer the effects of perceived stress on psychological functioning, decreasing psychological distress when under stress.^17^^,^^18^ Social support may facilitate the re-evaluation of stressful situations,^18^ and reduce the intensity of physiological stress responses through hormonal functioning.^19^^,^^20^ Prior research on the stress-buffering effects of social support in students has explored mostly overall perceived social support, focusing on support from family, romantic partners and friends.^21^^-^^26^However, only a few studies have explored the stress-buffering effects of source-specific social support,^27^ despite being essential for understanding what type of support students need during their education. Furthermore, while support from teachers has been associated with lower levels of perceived stress^28^^-^^30^and psychological distress in students^7^^,^^31^ the ability of teacher support to buffer the effects of perceived stress is yet to be explored.

In addition to dyadic relationships, larger social systems, such as social media and communities, may also have stress-buffering effects.^32^^,^^33^ Social media can offer information and other coping resources, such as peer support and opportunities to vent.^34^ Research on social media’s role in stress and coping is still sparse, but previous findings suggest that the use of social media may indeed buffer the effects of stress.^35^^-^^39^

Similarly, the student community can be a source of supportive and meaningful relationships with peers who share the stressors and experiences related to being a student, increasing the perception of available support.^32^^,^^40^^,^^41^ So far, only few studies have explored the role of communities in students’ perceived stress and psychological distress. These studies have found the student community to buffer the effects of perceived stress on life satisfaction and depressive symptoms, but not anxiety.^42^ However, the sense of community belonging, the feeling of being an important and valued member of a social system or a group^43^^,^^44^ could be a particularly important intervention target for students, as it appears relatively easy to improve.^45^^,^^46^

Moreover, the ability to find meaning in adversity can impact how stressful conditions affect people.^47^^-^^49^ Meaningfulness can motivate to take action to control the stressors^50^ and increase available personal resources, like hope and self-efficacy^51^ buffering the effects of perceived stress on psychological distress. Meaningfulness has been a topic of growing interest in various occupational fields, and studies on employees have found meaningful work to moderate the effects of perceived stress on different indicators of strain.^52^^,^^53^

Despite previous research on the stress-buffering effects of social support^21–26 ^less is known about the effects of source-specific support. Also, only limited research exists on the roles of teacher support, social media use and sense of community belonging in students’ perceived stress and psychological distress. Furthermore, to the best of our knowledge, the stress-buffering effects of meaningfulness of studying are yet to be investigated. Our study aims to add to the understanding of the supports healthcare students need, by exploring the relationship between perceived stress and two indicators of psychological distress, depressive symptoms and anxiety, and how social support from different sources (parents, partner, friends, peers, teachers, social media), sense of community belonging, and meaningfulness of studying impact these relationships.

## Methods

### Study design and participants

This cross-sectional study was conducted in 2018 in the Faculty of Medicine, University of Helsinki, Finland. It was part of a larger longitudinal study begun in 2017, aiming to gather information about the study progress and well-being of the students in the faculty. The target population of our study were all undergraduate and graduate students of medicine, dentistry, psychology and speech therapy in the Faculty of Medicine at the University of Helsinki.

Participants were sent an online survey via email. We received responses from 853 students, of which 53 were excluded from the study due to incomplete responses. We used a convenience sample representing 50% (n = 800) of the students in the faculty. The participants were between 18 to 40 years old, with a mean age of 25 years. Detailed characteristics of the participants are presented in Table 1.

Ethical approval was obtained from the Research Ethics Committee of the Faculty of Medicine, University of Helsinki, on 27.03.2017. Participation was voluntary, and all participants gave informed consent. The data was anonymized before the analyses, and the reporting ensured that the participants could not be identified.

**Table 1:** Sample demographic characteristics (N = 800)

Variable		N (%)
Subject		
	Medicine	392 (49)
	Dentistry	161 (20)
	Psychology	167 (21)
	Speech therapy	80 (10)
Gender		
	Female	580 (73)
	Male	212 (26)
	Other	6 (1)
First language		
	Finnish	693 (87)
	Swedish	87 (11)
	Other	17 (2)
Year of study		
	1	181 (23)
	2	154 (19)
	3	147 (18)
	4	132 (17)
	5	118 (15)
	6 or above	57 (7)
Parent with higher education		
	Mother	553 (69)
	Father	502 (63)

### Data collection

The data for our study were collected during the spring semester of 2018 using an online survey. The survey was sent out to all undergraduate and graduate students in the faculty via email. The survey included questions about demographic characteristics, study habits, personal tendencies, perceived stress, and health. Participants were informed that participation was voluntary and confidential. They were also informed about the purpose of the study.

### Instruments

#### Perceived stress

To measure perceived stress, we used an item from a Finnish work stress questionnaire widely used to assess perceived stress in many different populations.^54^ The item was a statement, “Stress refers to a situation wherein a person feels tense, restless, nervous, or anxious, or they have difficulty sleeping due to worrying. Think about the past month. Have you felt this kind of stress?”. It was rated on a 5-point scale (1 = “never”, 5 = “very much”).

#### Psychological distress

Psychological distress was assessed using the 15D Health-Related Quality of Life Questionnaire, an established measure of self-reported health with good reliability and validity.^55^ We used the items on depressive symptoms and anxiety, which have high sensitivity for generally well populations.^55^ The item on depressive symptoms was rated on a 5-point scale (1 =“I do not feel at all sad, melancholic, or depressed”, 5 = “I feel extremely sad, melancholic, or depressed”). The item on anxiety was rated on a 5-point scale (1 = “I do not feel at all anxious, stressed, or nervous”, 5 = “I feel extremely anxious, stressed, or nervous”).

#### Social support

The measure of social support was adapted for this study from the Social Support Questionnaire.^56^ The measure included seven sources of support (parents, partners, friends, peers, teachers, social media, student psychologist), of which student psychologist support was excluded due to being outside the scope of our study. Students were asked whom they would receive support from in four stressful study-related situations (e.g., when you fail an exam, when you are feeling overwhelmed). The items were rated on a 2-point scale (0 = “I would not receive support from this source in this situation”, 1 = “I would receive support from this source in this situation”). Sum variables were calculated from the four questions to represent the level of social support from each source, ranging from 0 to 4 with a higher score indicating a higher level of support. The Cronbach’s Alpha values were 0.89 for parent support, 0.96 for partner support, 0.86 for friend support, 0.88 for peer support, 0.68 for teacher support and 0.84 for social media support.

#### Sense of community belonging

Sense of community belonging was assessed with a question, “How often do you feel like you are a part of your study-related community?”. The item was rated on a 5-point scale (1 = “never”, 5 = “constantly”). This measure was adapted from the MED NORD instrument, developed, and validated to measure medical students’ well-being.^57^

#### Meaningfulness of studying

Meaningfulness of studying was assessed with the statement, “I have difficulty finding my studies meaningful”. The item was rated on a 5-point scale (1 = “never”, 5 = “constantly”), and was reversed for the analysis to be concordant with the other moderator variables. This measure was also adapted from the MED NORD instrument.^57^

#### Statistical analysis

Logistic regression analyses were conducted using the PROCESS Macro v. 4.0 (Model 1) to explore the relationship of perceived stress with depressive symptoms and anxiety, and to test if perceived stress would have significant interactions with parent support, partner support, friend support, peer support, teacher support, social media support, sense of community belonging and meaningfulness of studying in predicting depressive symptoms and anxiety. To further explore the moderator effects at different levels of the variables, conditional effects were calculated at the point of the mean +/-1 standard deviation. Confidence intervals (95%) were calculated to determine the level of significance using the bootstrapping method with 5000 iterations. Age, gender and subject were included as covariates. The data analysis was conducted using IBM SPSS Statistics version 27.

## Results

Descriptives and Pearson’s correlation coefficients between the study variables are presented in Table 2. All significant correlations were in the expected direction.

Perceived stress had a significant positive effect on depressive symptoms in all models, with the explained variance of the models ranging from 19% to 27% (Table 3). Perceived stress had significant interaction terms with parent support (B = -.03, t_(783)_ = -2.4, p < .001), partner support (B = -.05, t_(783)_ = -4.3, p < .001), peer support (B = -.04, t_(783)_  = -3.0, p < .001), sense of community belonging (B = -.06, t_(783)_ = -2.7, p < .01) and meaningfulness of studying (B = -.12, t_(783)_ = -4.5, p < .001) in predicting depressive symptoms. The results indicated that parent support, partner support, peer support, sense of community belonging and meaningfulness of studying moderated the effect of perceived stress on depressive symptoms, which was statistically significant at all three levels (mean +/- 1 SD) for all these variables, as the confidence intervals at 95% did not contain zero (Table 4).

**Table 2:** Descriptive Statistics and Pearson’s correlations coefficients for study variables (N = 800)

Items	M	SD	1	2	3	4	5	6	7	8	9	10	11
1. Perceived stress	3.4	0.49	-										
2. Depressive symptoms	1.6	0.62	.41^**^	-									
3. Anxiety	1.9	0.27	.57^**^	.57^**^	-								
4. Parent support	1.8	0.47	-0.06	-.19^**^	-.14^**^	-							
5. Partner support	2.3	6.62	-0.01	-.20^**^	-0.04	-0.05	-						
6. Friend support	2.1	6.98	-0.05	-.10^**^	-.09^*^	.33^**^	-0.04	-					
7. Peer support	2.6	1.5	-0.08	-.24^**^	-.20^**^	.28^**^	0.04	.34^**^	-				
8. Teacher support	0.1	0.5	.10^*^	0	0.05	0.04	0.01	.10^**^	.11^**^	-			
9. Social media support	0.1	0.6	0.02	-0.03	-0.02	0.04	0.05	.12^**^	.08^**^	.16^**^	-		
10. Sense of community belonging	3.7	1.1	-.12^**^	-.27^**^	-.24^**^	.20^**^	-0.01	.12^**^	.47^**^	0.07	.08^*^	-	
11. Meaningfulness of studying	4	0.8	-.23^**^	-.35^**^	-.31^**^	.08^*^	-.11^**^	.08^*^	.15^**^	0.05	-0.01	.29^**^	-
Age	24.8	4	.07^*^	0.01	0.01	-.20^**^	.15^**^	-.11^**^	-.08^*^	-0.01	-0.04	-.15^**^	-0.05
Gender			-.11^**^	0.06	-0.06	-.10^**^	-.09^*^	-.11^**^	-.10^**^	0.02	0	0.01	-0.01
Subject			.12^**^	.10^*^	.17^**^	-0.06	0.06	.11^**^	-.08^*^	0.01	-0.02	-0.16	0.07

^*^Significant at p<.05,^**^significant at p<.01

Perceived stress had a significant positive effect on anxiety in all models, with explained variance ranging from 35% to 38% (Table 5). Perceived stress had significant interaction terms with parent support (B = -.05, t_(783)_ = -3.8, p < .001), partner support (B = -.03, t_(783)_ = -2.2, p < .05), peer support (B = -.05, t_(783)_ = -3.5, p < .001), sense of community belonging (B = -.08, t_(783) _= -2.5, p < .01) and meaningfulness of studying (B = -.08, t_(783) _= -3.0, p < .01) in predicting anxiety. The results indicated that parent support, partner support, peer support, sense of community belonging and meaningfulness of studying moderated the effect of perceived stress on anxiety, which was statistically significant at all three levels (mean +/- 1 SD) for all these variables, as the confidence intervals at 95% did not contain zero (Table 6).

## Discussion

In this cross-sectional study of 800 medical, dental, psychology and speech therapy students, we aimed to examine the relationships between perceived stress and two indicators of psychological distress, depressive symptoms and anxiety, and the stress-buffering effects of different sources of social support, sense of community belonging and meaningfulness of studying. We found that higher perceived stress was associated with higher levels of both depressive symptoms and anxiety. These findings were consistent with previous studies^10–13^ indicating the importance of supporting healthcare students to manage the stressors of their education to prevent psychological distress and its consequences.^14–16^

Several sources of support, as well as the sense of community belonging and meaningfulness of studying, were found to buffer the effects of perceived stress on both depressive symptoms and anxiety. First, we found that perceived stress had a weaker effect on both depressive symptoms and anxiety among those who reported higher social support from parents and romantic partners. These findings support previous studies,^21–26^ except for support from friends, which did not have an impact on the relationship between perceived stress and psychological distress in our study.

Second, perceived stress had a weaker effect on psychological distress at higher levels of peer support and sense of community belonging. These findings also support previous studies,^42^^,^^22^ reflecting the importance of having supportive relationships with people who share the stressors and experiences of being a healthcare student. From a practical perspective, these findings are particularly significant, as peer support and the sense of community belonging could be intervention targets, that are both accessible to universities and also fairly responsive to intervention.^45^^,^^46^

**Table 3:** The moderating effects on the relationship between perceived stress (PS) and depressive symptoms

Moderating effects	PS coefficient [LLCI, ULCI]	Moderator coefficient [LLCI, ULCI]	Interaction coefficient [LLCI, ULCI]	Covariate coefficients [LLCI, ULCI]		F
Parent support	.29^***^ [.25, .34]	-.08^***^ [-.11, -.05]	-.03^***^ [-.06, .00]	Age	-.01 [-.02, .00]	36.43^***^
R^2^ = .22				Gender	.15^**^ [.04, .26]	
R^2^ change = .01^*^				Subject	.02^*^ [.00, .04]	
Partner support	.30^***^ [.25, .34]	-.08^***^ [-.11, -.05]	-.05^***^ [-.08, -.03]	Age	.00 [-.01, .01]	41.80^***^
R^2^ = .24				Gender	.17^***^ [.06, .28]	
R^2^ change = .02^***^				Subject	.03^**^ [.01, .05]	
Friend support	.30^***^ [.25, .35]	-.04^**^ [-.07, -.01]	.00 [-.03, .03]	Age	.00 [-.01, .01]	31.42^***^
R^2^ = .20				Gender	.17^***^ [.07, .28]	
R^2^ change = .00				Subject	.03^**^ [.01, .05]	
Peer support	.29^***^ [.24, .34]	-.09^***^ [-.13, -.06]	-.04^***^ [-.07, -.02]	Age	-.01 [-.02, .00]	39.06^***^
R^2^ = .23				Gender	.13^*^ [-.02, .00]	
R^2^ change = .01^***^				Subject	.02 [.00, .04]	
Teacher support	.31^***^ [.26, .36]	-.09 [-.22, .03]	.03 [-.10, .17]	Age	.00 [-.01, .01]	30.30^***^
R^2^ = .19				Gender	.19^***^ [.08, .30]	
R^2^ change = .00				Subject	.02^*^ [.00, .05]	
Social media support	.31^***^ [.26, .35]	-.04 [-.12, .04]	-.04 [-.12, .03]	Age	.00 [-.01, .01]	30.41^***^
R^2^ = .19				Gender	.19^***^ [.08, .30]	
R^2^ change = .00				Subject	.02^*^ [.00, .05]	
Sense of community belonging	.29^***^ [.25, .34]	-.15^***^ [-.19, -.11]	-.06^**^ [-.10, -.02]	Age	-.01 [-.02, .00]	41.40^***^
R^2^ = .24				Gender	.16^***^ [.05, .27]	
R^2^ change = .01^**^				Subject	.01 [-.01, .03]	
Meaningfulness of studying	.27^***^ [.22, .31]	-.21^***^ [-.27, -.15]	-.12^***^ [-.17, -.07]	Age	-.00 [-.01, .01]	48.08^***^
R^2 ^= .27				Gender	.15^**^ [.04, .01]	
R^2^ change = .02^***^				Subject	.02 [.00, .04]	

PS: perceived stress, LLCI: lower limit confidence interval (95%), UCLI: upper limit confidence interval (95%) ^*^significant at p<.05,^**^significant at p<.01, ^***^significant at p<.001

**Table 4:** Conditional effects on depressive symptoms

Moderator		Effect	SE	t	LLCI	UCLI
Parent support	-1SD	0.35	0.03	10.9^***^	0.28	0.41
	M	0.29	0.02	12.5^***^	0.25	0.34
	+1SD	0.24	0.03	6.9^***^	0.17	0.31
Partner support	-1SD	0.4	0.03	12.5^***^	0.34	0.46
	M	0.3	0.02	12.9^***^	0.25	0.34
	+1SD	0.21	0.03	6.6^***^	0.14	0.27
Peer support	-1SD	0.36	0.03	11.3^***^	0.3	0.42
	M	0.29	0.02	12.5^***^	0.24	0.34
	+1SD	0.23	0.03	7.4^***^	0.17	0.29
Sense of community belonging	-1SD	0.36	0.03	10.8^***^	0.29	0.42
	M	0.29	0.02	12.6^***^	0.25	0.34
	+1SD	0.23	0.03	7.1^***^	0.16	0.29
Meaningfulness of studying	-1SD	0.36	0.03	11.3^***^	0.2	0.43
	M	0.27	0.02	11.5^***^	0.22	0.31
	+1SD	0.17	0.03	5.5^***^	0.11	0.23

SD: standard deviation, M: mean, SE: standard error, LLCI: lower limit confidence interval (95%), UCLI: upper limit confidence interval (95%),

^***^significant at p<.001

**Table 5:** The moderating effects on the relationship between perceived stress and anxiety

Moderating effects	PS coefficient [LLCI, ULCI]	Moderator coefficient [LLCI, ULCI]	Interaction coefficient [LLCI, ULCI]	Covariate coefficients [LLCI, ULCI]		F
Parent support	.44^**^^*^ [.39, .48]	-.06^***^ [-.08, -.03]	-.05^***^ [-.07, -.02]	Age	-.01 [-.02, .00]	76.05^***^
R^2 =^ .37				Gender	.01 [-.10, .11]	
R^2 ^change = .01^***^				Subject	.03^***^ [.01, .06]	
Partner support	.45^***^ [.40, .49]	-.02 [-.04, .01]	-.03^*^ [-.05, .00]	Age	-.01 [-.02, .00]	70.59^***^
R^2 =^ .35				Gender	.03 [-.07, .14]	
R^2 ^change = .00^*^				Subject	.04^***^ [.02, .06]	
Friend support	.44^**^^*^ [.40, .49]	-.04^**^ [-.07, -.01]	-.01 [-.04, .02]	Age	-.01 [-.02, .00]	70.89^***^
R^2 =^ .35				Gender	.02 [-.09, .13]	
R^2 ^change = .00				Subject	.04^***^ [.02, .06]	
Peer support	.44^**^^*^ [.39, .48]	-.08^***^ [-.11, -.05]	-.05^***^ [-.08, -.02]	Age	-.01 [-.02, .00]	78.53^***^
R^2 =^ .38				Gender	-.02 [-.12, .09]	
R^2 ^change = .01^***^				Subject	.03^***^ [.01, .05]	
Teacher support	.45^***^ [.41, .50]	-.08 [-.20, .04]	.12 [-.02, .25]	Age	-.01 [-.02, .00]	69.78^***^
R^2 =^ .35				Gender	.04 [-.07, .14]	
R^2 ^change = .00				Subject	.04^***^ [.02, .06]	
Social media support	.45^***^ [.40, .49]	-.03 [-.11, .05]	-.01 [-.08, .06]	Age	-.01 [-.02, .00]	69.13^***^
R^2 =^ .35				Gender	.03 [-.07, .14]	
R^2 ^change = .00				Subject	.04^***^ [.02, .06]	
Sense of community belonging	.44^**^^*^ [.39, .48]	-.13^***^ [-.17, -.08]	-.05^**^ [-.09, -.01]	Age	-.01^*^ [-.02, .00]	79.93^***^
R^2 =^ .38				Gender	.01 [-.10, .11]	
R^2 ^change = .01^**^				Subject	.03^**^ [.01, .05]	
Meaningfulness of studying	.42^***^ [.37, .46]	-.17^***^ [-.23, -.11]	-.08^**^ [-.13, -.03]	Age	.00 [-.01, .01]	81.83^***^
R^2 =^ .38				Gender	.00 [-.10, .11]	
R^2 ^change = .01^**^				Subject	.03^***^ [.01, .06]	

**Table 6:** Conditional effects on anxiety

Moderator	Condition	Effect	SE	t	LLCI	UCLI
Parent support	-1SD	0.52	0.03	16.8^***^	0.46	0.58
	M	0.44	0.02	19.1^***^	0.39	0.48
	+1SD	0.35	0.03	10.5^***^	0.29	0.42
Partner support	-1SD	0.50	0.03	15.5^***^	0.43	0.56
	M	0.45	0.02	19.3^***^	0.40	0.49
	+1SD	0.40	0.03	12.7^***^	0.34	0.46
Peer support	-1SD	0.51	0.03	16.5^***^	0.45	0.57
	M	0.44	0.02	19.2^***^	0.39	0.48
	+1SD	0.37	0.03	12.2^***^	0.31	0.43
Sense of community belonging	-1SD	0.49	0.03	15.3^***^	0.43	0.56
	M	0.44	0.02	19.3^***^	0.39	0.48
	+1SD	0.38	0.03	12.1^***^	0.32	0.44
Meaningfulness of studying	-1SD	0.48	0.03	15.0^***^	0.42	0.55
	M	0.41	0.02	18.1^***^	0.37	0.46
	+1SD	0.35	0.03	11.6^***^	0.29	0.41

SE: standard error, LLCI: lower limit confidence interval (95%), UCLI: upper limit confidence interval (95%), ***significant at p<.001

Third, perceived stress also had a weaker effect on psychological distress among students who reported higher meaningfulness of studying. To the best of our knowledge, there are no previous studies on the stress-buffering effects of the meaningfulness of studying. However, previous studies have found meaningful work to moderate the effects of perceived stress on different indicators of strain.^52^^,^^53^ Our result supports these findings, suggesting a similar protective effect of meaningfulness for students as well. Certain stressors are unavoidable when pursuing healthcare education,^6^^-^^9^but the stressors may be easier to tolerate when they feel meaningful.

Finally, no stress-buffering effects were found for teacher support, despite having been associated with lower levels of both stress ^28^^-^^30^and psychological distress in students.^7^^,^^31^ The levels of teacher support were also very low on average, suggesting that the students did not find it natural to turn to their teachers in stressful situations. Similarly, social media support did not buffer the effects of perceived stress, contrary to previous findings.^35^^-^^39^However, we approached the use of social media from a different perspective than previous studies, asking in which study-related struggles the student might utilize social media for support, as opposed to investigating the number of social media contacts or social media behaviors. If social media is used to manage stress, it might not be done intentionally, i.e., people do not explicitly seek support from social media but rather receive it through their online behaviors.

There is a high prevalence of perceived stress and psychological distress among healthcare students^15^^-^^4^which can have negative implications for students’ academic performance and increase the risk for future ill-being and burnout.^14^^-^^16^To combat this, the findings of our study suggest that students should be encouraged to identify their sources of support and seek support from those sources in stressful situations. However, not all students have supportive relationship in their life outside of the university setting. Thus, it is particularly important to promote students’ sense of community belonging and provide opportunities for students to connect with their peers, both inside and outside of the classroom. Formal peer support programs have also yielded positive results on student well-being.^58^ Furthermore, educators and student welfare providers should consider strategies for promoting the meaningfulness of studying in healthcare students.

### Limitations and future research

Despite the strengths of our study, which include a large sample size of 800 participants and the use of established measures, as well as the multi-dimensional approach to social support, there are also limitations to consider. First, our results are not generalizable to other healthcare student populations, as we used a convenience sample with participants from only one university. Second, there may have been selection bias despite the 50% response rate. For example, our participants reported fairly low levels of depressive symptoms and anxiety, contrary to previous research findings,^1^^-^^4^ possibly indicating that the most distressed students did not participate. Third, as we used a self-administered survey, they may have been social desirability bias, despite the measures taken to ensure the anonymity of the participants. Fourth, our data was from 2018 and therefore does not account for the changes in higher education or social media use there has been in recent years. Finally, our study was cross-sectional, and thus no inferences of the direction or causality between the study variables can be made.

Future research is needed to establish the stress-buffering effects found in our study with longitudinal study designs and to further explore the role of teacher support and social media use in healthcare students’ perceived stress and psychological distress. Further research is also needed on factors predicting the meaningfulness of studying.

## Conclusions

Our study found that perceived stress predicted depressive symptoms and anxiety in healthcare students. Social support from parents, romantic partners and peers, as well as the sense of community belonging and meaningfulness of studying, were found to buffer the effects perceived stress had on psychological distress. These findings offer valuable information for healthcare educators and student welfare providers who seek to promote students’ well-being.

### Acknowledgments

The authors would like to acknowledge Ms. Tiina Härkönen for assistance in coordination, and former dean Professor Risto Renkonen for his support for the student well-being project.

### Conflicts of Interest

The authors declare that they have no conflicts of interest.
